# Anesthesia for endovascular treatment in anterior circulation stroke: A systematic review and meta‐analysis

**DOI:** 10.1002/brb3.1178

**Published:** 2018-12-03

**Authors:** Xuefei Li, Zheng Hu, Qian Li, Yinping Guo, Shabei Xu, Wei Wang, Dan He, Xiang Luo

**Affiliations:** ^1^ Department of Neurology, Tongji Hospital, Tongji Medical College Huazhong University of Science and Technology Wuhan China; ^2^ Department of Obstetrics and Gynecology, The first Affiliated Hospital Sun Yat‐sen University Guangzhou China; ^3^ Department of Neurology Shenzhen Shekou People’s Hospital Shenzhen China; ^4^ Department of Neurology, National Key Clinical Department and Key Discipline of Neurology, The First Affiliated Hospital Sun Yat‐sen University Guangzhou China

**Keywords:** acute ischemic stroke, anterior circulation, conscious sedation, endovascular treatment, general anesthesia, meta‐analysis

## Abstract

**Background:**

Endovascular treatment in patients with acute anterior circulation stroke could be performed under either conscious sedation (CS) or general anesthesia (GA). Although several studies have investigated the association between the clinical outcomes and the two anesthesia methods, consensus is lacking.

**Methods:**

PubMed and EMBASE searches were used to select full‐text articles comparing the effects of GA and CS on functional outcome and complications in patients with anterior circulation ischemic stroke. Enrolled patients were assigned to receive endovascular treatment with CS or GA, with a primary outcome of functional independency within 90 days. Secondary outcomes included intracranial hemorrhage, all‐cause mortality at 90 days, pneumonia, and intraprocedural complications.

**Results:**

Thirteen studies (3 RCTs and 10 observational studies), which included 3,857 patients (CS = 2,129, GA = 1,728), were eligible for the analysis. The overall analysis including the RCTs and observational studies demonstrated that the functional independence within 90 days occurred more frequently among patients with CS compared with GA (OR, 1.42; 95% CI, 1.05–1.92, *p* = 0.02); and the risk of mortality was higher with GA compared with CS; furthermore, CS was associated with lower rate of intracranial hemorrhage. In RCTs, GA was associated with increased functional independence (OR, 0.55; 95% CI, 0.34–0.89, *p* = 0.01) and successful reperfusion (OR, 0.51; 95% CI, 0.30–0.89, *p* = 0.02).

**Conclusions:**

In the overall analysis and observational studies, CS was associated with improved functional outcomes and relatively safe for anterior ischemic stroke compared with GA. While the pooled data from RCTs suggested that GA was associated with improved outcomes. The inconsistency indicated that more large‐scale RCTs are required to evaluate what factors influenced the effect of the anesthesia methods on clinical outcomes.

## 1 INTRODUCTION

Endovascular treatment (EVT) with mechanical thrombectomy is safe and effective in patients with acute anterior circulation stroke, compared with intravenous tissue plasminogen activator (IV‐tPA) (Badhiwala et al., 2015; Elgendy, Kumbhani, Mahmoud, Bhatt, & Bavry, 2015; Kim, Jeon, Kim, Choi, & Cho, 2018; Marmagkiolis et al., 2015). However, the primary clinical outcomes are affected by many factors, such as the site of occlusion, stroke severity, and patient management factors including blood pressure during thrombectomy (Adams et al., 2007; Hungerford et al., 2016). Previous research has demonstrated that the anesthesia types would also impact the hemodynamic change (Jagani, Brinjikji, Rabinstein, Pasternak, & Kallmes, 2016), thereby influencing the outcomes of endovascular therapy. Currently used anesthetic techniques primarily include conscious sedation (CS) and general anesthesia (GA). However, there is a debate over which type of anesthesia is more beneficial to patients. During endovascular treatment with GA, the airway is more protected, and the intraprocedural complications are less observed due to patient immobility (Li et al., 2014; Slezak et al., 2017). The unfavorable hemodynamic changes including hypotension and treatment delay are potential disadvantages of GA (Jagani et al., 2016). While the advantages of CS include that interventionalists can continuously monitor patient neurological functions during the procedure and the duration of time to complete endovascular treatment can be reduced (Li et al., 2014).

Previous retrospective studies comparing anesthesia methods during mechanical thrombectomy for anterior circulation ischemic stroke have concluded that CS is preferable to GA (Slezak et al., 2017; Whalin et al., 2014; Berg et al., 2015; Berkhemer et al., 2016; Jumaa et al., 2010; Abou‐Chebl et al., 2014; Abou‐Chebl et al., 2010; John et al., 2014; Nichols et al., 2010), other than one study (Bracard et al., 2016) which found that there was no difference in the functional independence of the two anesthesia methods. Currently, three RCTs compared the clinical outcomes of the various anesthesia methods for anterior circulation ischemic stroke, one of which, ANSTROKE (Löwhagen Hendén et al., 2017) showed that the clinical outcomes of the two anesthetic techniques were similar. The other two RCTs, SIESTA (Schönenberger et al., 2016) and GOLIATH (Simonsen et al., 2018), demonstrated that GA did not result in worse clinical outcomes compared with CS. Although a meta‐analysis, Ilyas et al., (2018) found that there was no significant difference between the CS group and GA group for acute anterior circulation ischemic stroke using Solitaire stent retriever, there were some limitations such as few studies and the results from a mixture of prospective and retrospective studies. Furthermore, no meta‐analysis has separately analyzed the current data of RCTs and observational studies for anterior circulation ischemic stroke. Therefore, we performed a meta‐analysis of complete results from RCTs and observational studies to evaluate the association between the clinical outcomes and the anesthesia types during endovascular treatment for anterior circulation ischemic stroke.

## 2 MATERIALS AND METHODS

### 2.1 Search strategy and selection criteria

The major online databases, PubMed and EMBASE, were searched to identify the comparative studies on CS versus GA during endovascular treatment for acute anterior circulation ischemic stroke, from inception to January 2018, using the Medical Subject Heading (MeSH) terms and the keywords as follows: (a) the terms pertinent to the anesthesia methods including general anesthesia, conscious sedation, and local anesthesia; (b) the terms pertinent to the intervention of interest including endovascular, thrombectomy, intra‐arterial, thromboembolism, fibrinolysis, and thrombolysis; and (c) the terms pertinent to the patient conditions including anterior circulation, ischemic, stroke, cerebrovascular accident, and infarct. The search terms were used in relevant combinations. In addition, previous systematic reviews and meta‐analyses related to anesthesia management during mechanical thrombectomy were critically reviewed (Brinjikji et al., 2015; Campbell et al., 2018; Erickson & Cole, 2005; Ilyas et al., 2018; John, Mitchell, Dowling, & Yan, 2013).

The following inclusion criteria for this meta‐analysis were used: (a) the studies that only included anterior circulation infarct; (b) the articles that compared the clinical results of CS with that of GA during endovascular treatment; (c) the researches that reported the modified Rankin scale (mRS) at 90 days in both CS and GA groups. We also included post hoc analyses (Abou Chebl et al., 2015; Berg et al., 2015; Nichols et al., 2010) except for one study, Pfaff JAR et al (Pfaff et al., 2018), the results of which were duplicate with that of the SIESTA (Schönenberger et al., 2016). We excluded duplicate reports, abstracts that were not published as full‐text reports in a journal and articles without mRS at 90 days in both CS and GA groups. Moreover, studies that included GA or CS only and studies that reported the posterior circulation stroke were also excluded. Two investigators independently examined each study to determine whether to be included or excluded based on the selection criteria. Disagreements between the two investigators were resolved by a third investigator. All researches obtained ethics approval from the local institutional boards at participating sites. Although this meta‐analysis was not registered, the Preferred Reporting Items for Systematic Reviews and Meta‐Analyses (PRISMA) (Moher, Liberati, Tetzlaff, & Altman, 2009) were followed.

### 2.2 Quality assessment

Two reviewers independently used the Cochrane Collaboration's tool to assess the risk of selection bias, performance bias, detection bias, attrition bias, reporting bias, and other sources of bias (Higgins et al., 2011) among the RCTs. We assessed the quality of the observational studies using the Newcastle–Ottawa Scale (NOS), including selection, comparability, and outcomes (Stang, 2010).

### 2.3 Data extraction and outcome definitions

Data were independently extracted by two investigators. The following characteristics were examined: (a) descriptive summary of each study (study name, author, year of publication, and total number of patients) and (b) patient characteristics (age, hypertension, atrial fibrillation, hyperlipidemia, diabetes mellitus, smoking, site of occlusion, and baseline NIHSS).

Our prespecified clinical endpoints included both primary and secondary outcomes. The primary outcome was functional independence, as defined by mRS scores (from 0 to 6) of 0–2 within 90 days. Our secondary efficacy outcome was the proportion of patients with successful revascularization indicated by a modified thrombolysis in cerebral infarction (mTICI) score ≥2b (perfusion with distal branch filling ≥50%). Our secondary safety outcomes were intracranial hemorrhage (as defined by each trial), all‐cause mortality at 90 days, pneumonia, and intraprocedural complications (including device‐related complications, vessel perforation, dissection, and groin hematoma).

### 2.4 Statistical analyses

The extracted data were analyzed by meta‐analysis software including STATA version 11 (StataCorp, College Station, Texas, USA) and Review Manager (RevMan) version 5.3. Characteristics of patients are presented as numbers and percentages for categorical variables, and continuous data were expressed as means ± standard deviations (*SD*s). When the median, range, and sample size were provided, we estimated the mean and variance according to a formula (Hozo, Djulbegovic, & Hozo, 2005). Mean differences (*MD*s) and 95% confidence intervals (CIs) were calculated for pooled continuous variables. Random‐effects summary odds ratios (ORs) with corresponding 95% CIs were also constructed for the prespecified primary and secondary clinical endpoints, using RevMan with the DerSimonian and Laird random‐effects model (DerSimonian & Laird, 2015).

Statistical heterogeneity was assessed by the *I* ^2^ statistic, with values <25%, 25%–50%, and >50% as low, moderate, and high degree of heterogeneity, respectively (Higgins, Thompson, Deeks, & Altman, 2003). To further estimate heterogeneity of the primary outcome, we performed subgroup and sensitivity analyses and meta‐regressions. Funnel plots were used to visually evaluate publication bias, and Egger regressions were simultaneously used for quantification (Egger, Davey Smith, Schneider, & Minder, 1997). A two‐tailed value of *p*＜0.05 was considered statistically significant.

## 3 RESULTS

**Figure 1:**
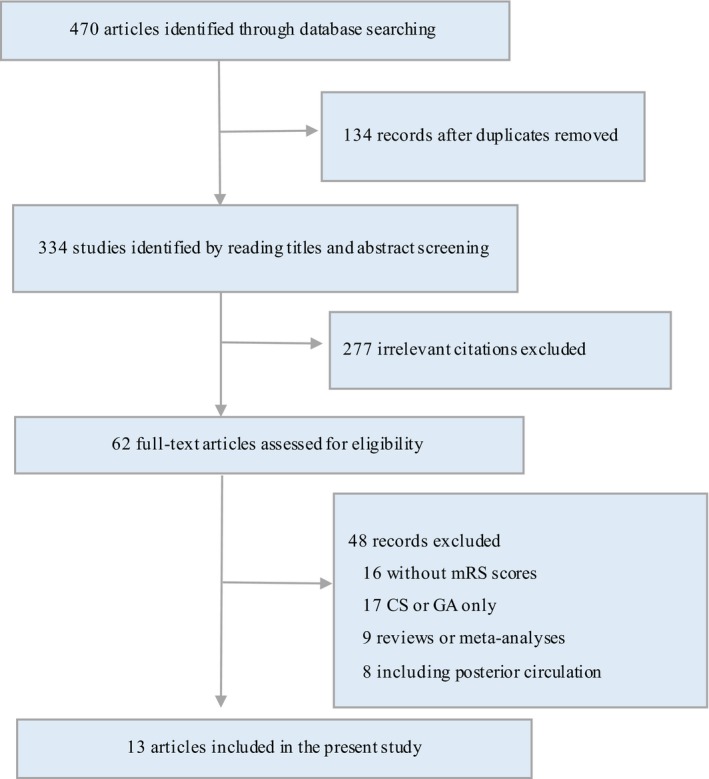
Flowchart of literature screening. mRS, modified Rankin Scale

Figure 1 shows that our search strategy finally incorporates 13 studies (3 RCTs (Löwhagen Hendén et al., 2017; Schönenberger et al., 2016; Simonsen et al., 2018) and 10 observational studies (Abou‐Chebl et al., 2010; Abou‐Chebl et al., 2014; Berg et al., 2015; Bracard et al., 2016; John et al., 2014; Jumaa et al., 2010; Shan et al., 2018; Slezak et al., 2017; Whalin et al., 2014)) available for analysis. A total of 2,129 patients underwent endovascular therapy for anterior ischemic stroke by CS, while 1,728 patients by GA. The characteristics of studies were summarized in Table 1. All RCTs were single‐center randomized trials, which were published between 2016 and 2018. The publication dates of the observational studies ranged from 2010 to 2018. All except three studies (Abou‐Chebl et al., 2014; Bracard et al., 2016; Nichols et al., 2010) reported the baseline NIHSS scores between CS and GA, and the baseline NIHSS scores were lower in the CS group compared with the GA group in five studies (Abou‐Chebl et al., 2010; Jumaa et al., 2010; Löwhagen Hendén et al., 2017; Slezak et al., 2017; Whalin et al., 2014).

**Table 1:** Descriptive summary of patients and study characteristics

Author (year)	No. of patients	Observational studies included in the meta‐analysis									
		Type of anesthesia									
		CS					GA				
		No. of patients	Stroke location	Intervention methods *n* (%)	Age (mean, *SD*)	Baseline NIHSS (mean, *SD*)	No. of patients	Stroke location	Intervention methods *n* (%)	Age (year)	Baseline NIHSS (mean, *SD*)
van den Berg et al. (2015)	348	278	AC278	EVT61(21.9) IV‐tPA 81(29.1) EVT+IV‐tPA 136 (48.9)	62 (14.0)	15 (7.0)	70	AC70	EVT32(45.7) IA‐tPA 9(12.9); EVT+IA‐tPA 29 (41.4)	57 (17.7)	16 (5.0)
Slezak et al. (2017)	401	135	AC135	EVT 72 (53.3) EVT+IV‐tPA 44 (32.6)	70.5 (14.9)	13.1 (5.7)	266	AC266	EVT159(59.8) EVT+IV‐tPA 78 (29.3)	70.9 (13.9)	17.2 (6.6)
Whalin et al. (2014)	216	83	AC83	EVT39(47.0) EVT+IV‐tPA 44(53.0)	68.2 (15.3)	17 (3.4)	133	AC133	EVT63(47.4) EVT+IV‐tPA 70(52.6)	63.5 (13.6)	20 (2.0)
Jumaa et al. (2010)	126	73	AC73	EVT53(72.6) EVT+IV‐tPA 20(27.4)	66.6	15.1 (2.0)	53	AC53	EVT33(62.3) EVT+IV‐tPA 20(37.7)	66.5	17.6 (4.0)
Abou‐Chebl et al. (2015)	252	82	AC82	NS	69 (15.6)	NS	170	AC170	NS	66.4 (14.8)	NS
Alex Abou‐Chebl et al. (2010)	980	552	AC552	NS	NS	16 (6)	428	AC428	NS	NS	17 (5)
John et al. (2014)	190	99	AC99	EVT60(60.6) EVT+IV‐tPA 39(39.4)	69.1 (13.5)	15.4 (6.4)	91	AC91	EVT62(68.1) EVT+IV‐tPA 29(31.9)	64.8 (17.0)	16.3 (6.8)
Barcard et al. (2016)	141	74	AC74	NS	NS	NS	67	AC67	NS	NS	NS
HERMES, ISC. (2017)	609	456	AC456	NS	NS	NS	153	AC153	NS	NS	NS
Shan et al. (2018)	228	1,114	AC114	NS	65.5 (3.3)	16 (2.0)	114	AC114	NS	63.5 (5.0)	16 (2.5)
Randomized controlled trials included in the meta‐analysis											
Schönenbergr et al. (2016)	150	77	AC77	EVT27 (35.1); EVT+IV‐tPA 50 (64.9)	71.2 (14.7)	16.8 (7.8)	73	AC73	EVT26(35.6); EVT+IV‐tPA 46(63.0); None1 (1.4)	71.8 (12.9)	17.2 (7.4)
LöwhagenHendén et al. (2017)	90	45	AC45	EVT9(20.0) EVT+IV‐tPA 36(80.0)	72 (8.0)	17 (3.2)	45	AC45	EVT12(26.7) EVT+IV‐tPA 33(73.3)	73 (7.5)	20 (3.5)
Simonsen et al. (2018)	128	63	AC63	EVT+IA‐ tPA 8(12.7) EVT+IV‐tPA 46(73.0)	71.8 (12.8)	17 (2.0)	65	AC65	EVT+IA‐ tPA 9(13.8) EVT+IV‐tPA 50(76.9)	71.0 (10.0)	18 (2.6)

AC: anterior circulation; CS: conscious sedation; EVT: endovascular treatment; GA: general anesthesia; IA‐tPA: intra‐arterial tissue plasminogen activator; IV‐tPA: intravenous tissue plasminogen activator; NIHSS: The National Institutes of Health Stroke Scale; NS: not specified. Data are *n* (%) or means (±*SD*s, standard deviations).

To evaluate the features of patients in the GA and CS groups, we analyzed the demographic data (Supporting information Table S1). In the overall analysis, hypertension and heart disease were more frequently observed in patients with GA (*p* = 0.029, 0.008, respectively). For the site of occlusion, the rates of the internal cerebral artery (ICA, *p* = 0.000) were higher for GA, whereas the percentage of the middle cerebral artery (MCA) were lower (*p* = 0.000), compared with CS. The mean baseline NIHSS score was lower in patients with CS (MD −1.86, *p* = 0.000). Additionally, the mean duration from symptom onset to endovascular treatment was longer for patients who experienced GA than CS (MD −11.57, *p* = 0.003). However, there were no significant differences in other characteristics between two groups. The results of observational studies were consistent with the overall analysis except that hyperlipidemia was more frequent in patients receiving GA during endovascular treatment (*p* = 0.038). For the RCTs, the mean baseline NIHSS score was still lower in CS group, while other factors including the mean duration from symptom onset to mechanical thrombectomy were shown no substantial differences between two groups (Supporting information Table S1). Overall, risk of bias was rated as low for RCTs, as assessed by the Cochrane Collaboration's tool (Supporting information Figure S1) and Newcastle–Ottawa Scale quality scores were at least 7 stars for the observational studies, indicating high quality, except for three studies (Jumaa et al., 2010, Abou‐Chebl et al., 2015, and John et al., 2014 with 4 stars, revealing middle quality).

### 3.1 Primary outcome

**Figure 2:**
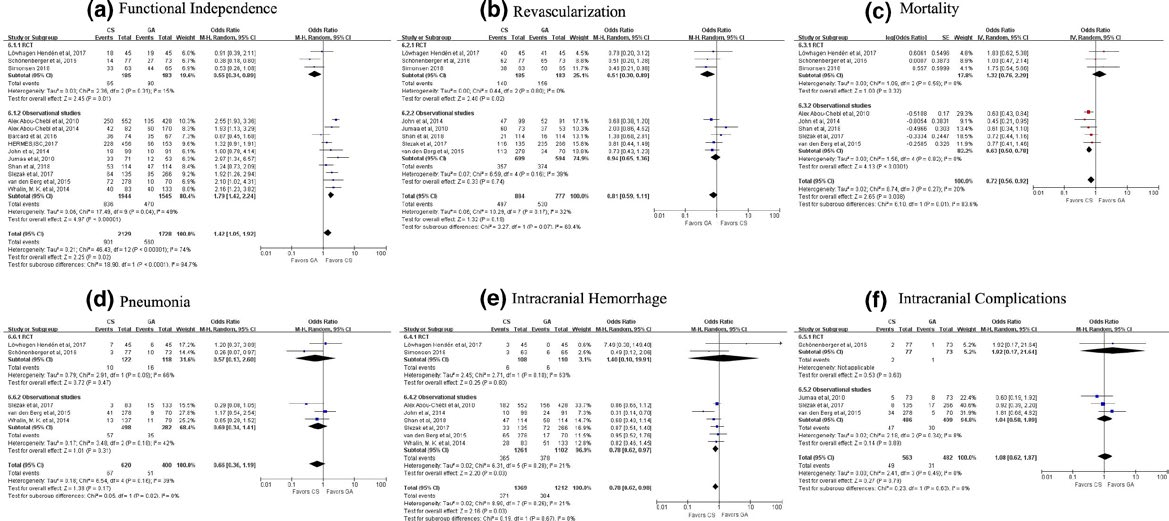
(a), Forest plot for primary outcome in the all studies. (b), (c), (d), (e), and (f), Forest plots for secondary efficacy and safety outcomes of conscious sedation (CS) versus general anesthesia (GA). Secondary clinical endpoints, including revascularization at the end of endovascular therapy, mortality at 90 days, pneumonia, intracranial hemorrhage, and intraprocedural complications in the overall analysis, observational studies, and RCTs, respectively

Compared with GA, the pooled data from 13 studies indicated that patients receiving CS had higher rates of functional independence within 90 days (OR, 1.42; 95% CI, 1.05–1.92, *p* = 0.02; *I* ^2^ = 74%; Figure 2a). For the observational studies, the primary outcome was in accordance with the total combined effect (OR, 1.79; 95% CI, 1.42–2.24; *p* ＜ 0.0001, *I* ^2^ = 49%, Figure 2a and Supporting information Table S4). For the RCTs, GA was associated with significantly higher rate of functional independence than CS (OR, 0.55; 95% CI, 0.34–0.89, *p* = 0.01) by random‐effects models with low heterogeneity (*I* ^2^ = 15%, Figure 2a and Supporting information Table S4), which was in opposite to the result of observational studies.

There was no apparent systematic bias of the primary clinical endpoint, as assessed by funnel plots (Supporting information Figure S2) and Egger regressions, for all included studies, observational studies, or RCTs (Egger test, *p* = 0.564, 0.976, and 0.760, respectively, in Supporting information Table S5). Furthermore, we conducted subgroup and sensitivity analyses to examine the relative efficacy of CS versus GA, stratified by the following prespecified variables: the baseline NIHSS scores, number of enrolled patients, age, ASPECT scores before treatment, time from onset to endovascular therapy, the usage of IV‐tPA, the thrombectomy devices, and the study with the risk of bias. There was no evidence of treatment heterogeneity effects for any of the prespecified variables (Table 2, *p* interaction > 0.05). Meta‐regression was further conducted to assess heterogeneity for the functional independence in all studies and the observational studies, adjusting for number of enrolled patients, and time from onset to mechanical thrombectomy. The adjusted ORs for the functional independence in the observational studies were 1.67 and 1.72, respectively, with no evidence of heterogeneity (*p* for heterogeneity > 0.05, Supporting information Table S3). Data of NIHSS scores and ASPECT scores were insufficient, so we did not conduct meta‐regressions. Furthermore, there were only 3 RCTs, and there were no significant differences in the pooled demographic characteristics other than NIHSS scores; therefore, meta‐regressions were not performed.

**Table 2:** Subgroup and sensitivity analyses for functional independence across observational studies

	NIHSS scores		Confirmed IV‐tPA		No. of patients			Study with risk of bias		
	13–16.5	16.6–20	Yes	No	＜200	200–299	≥300	High	Middle	Low
CS patients	437/1,135 (38.5%)	92/220 (41.8%)	279/803 (34.7%)	292/634 (46.1%)	51/170 (30.0%)	134/302 (44.4%)	386/965 (40.0%)	30/49 (61.2%)	93/252 (36.9%)	688/1578 (43.6%)
GA patients	20/161 (12.4%)	260/959 (27.1%)	175/692 (25.3%)	165/598 (27.6%)	22/144 (15.3%)	118/382 (30.9%)	200/764 (26.2%)	6/26 (23.1%)	82/314 (26.1%)	330/1,117 (29.5%)
Odds ratio (95%CI)	4.41 (2.72, 7.16)	1.93 (1.43, 2.62)	2.08 (1.63, 2.67)	2.40 (1.88, 3.07)	2.34 (1.32, 4.15)	2.04 (1.47, 2.85)	2.32 (1.86, 2.89)	5.26 (1.79, 15.47)	1.65 (1.16, 2.37)	1.84 (1.57, 2.17)
*p* value	＜0.00001	0.001	＜0.00001	＜0.00001	0.004	＜0.001	＜0.00001	0.003	0.006	＜0.00001
*I* ^2^	0%	0%	0%	60%	0%	0%	0%	0%	0%	0%
*p* interaction	0.50		0.10		0.82			0.48		

	Age(year)		ASPECT scores		Time to start EVT (min)		Thrombectomy devices	
	≥70	＜70	Yes	No	≥300	＜300	Modern	Premodern
CS patients	64/135 (47.4%)	182/597 (30.5%)	70/236 (29.7%)	501/1,201 (41.7%)	75/153 (49.0%)	228/633 (36.0%)	142/291 (48.8%)	283/623 (45.4%)
GA patients	85/266 (32.0%)	78/373 (20.9%)	28/170 (16.5%)	312/1,120 (27.9%)	112/356 (31.5%)	113/415 (27.2%)	180/503 (35.8%)	117/481 (24.3%)
Odds ratio (95%CI)	1.92 (1.26, 2.94)	2.06 (1.48, 2.88)	1.97 (1.19, 3.25)	2.28 (1.89, 2.75)	2.09 (1.42, 3.09)	1.50 (1.15, 1.97)	1.71 (1.28, 2.29)	2.59 (1.99, 3.36)
*p* value	0.003	＜0.001	0.008	＜0.001	0.0002	0.002	0.0003	＜0.001
*I* ^2^	‐	0%	0%	0%	0%	0%	0%	0%
*p* interaction	0.80		0.59		0.67		0.65	

ASPECT, Alberta Stroke Program Early Computed Tomography; CI, confidence interval; CS, conscious sedation; EVT, endovascular treatment; GA, general anesthesia; IV‐tPA, intravenous tissue plasminogen activator; NIHSS, The National Institutes of Health Stroke Scale.

### 3.2 Secondary outcomes

The secondary clinical endpoints in the present study included efficacy and safety outcomes in both groups (Figure 2). For the overall analysis, there was no significant difference between CS and GA in the rate of successful angiographic revascularization, defined as secondary efficacy outcome (OR, 0.81; 95% CI, 0.59–1.11; *p* = 0.19). The percentages of mortality and intracranial hemorrhage were significantly lower with CS when compared with GA (OR, 0.72; 95% CI, 0.56–0.92; *p* = 0.008; OR, 0.78; 95% CI, 0.62–0.97; *p* = 0.03, respectively). Other clinical outcomes were nonsignificant different between the two groups. There was no significant heterogeneity detected in data.

For the RCTs, GA was associated with higher rate of successful revascularization (OR, 0.51; 95% CI, 0.30–0.89; *p* = 0.020). There were no differences in the rates of all‐cause mortality at 90 days, intracranial hemorrhage, or intraprocedural complications between CS and GA (OR, 1.30; 95% CI, 0.76–2.22; *p* = 0.330; OR, 0.57, 95% CI, 0.13–2.60, *p* = 0.470; OR, 1.40, 95% CI, 0.10–19.91, *p* = 0.800; and OR, 1.92, 95% CI, 0.17–21.64, *p* = 0.600, respectively, Supporting information Table S4).

For the observational studies, the risk of all‐cause mortality and intracranial hemorrhage were lower in the CS group (OR, 0.63; 95% CI, 0.50–0.78; *p* = 0.000; OR, 0.78, 95% CI, 0.62–0.97, *p* = 0.03), which were in accordance with that of the overall analysis (Figure 2 and Supporting information Table S4). Other clinical endpoints were similar between two groups. There was no significant heterogeneity in any endpoints other than revascularization (*I* ^2^ = 63%, *p* = 0.01, Supporting information Table S4).

There was no evidence of systematic bias, as visually assessed by funnel plots and quantitatively assessed by Egger tests, with details as follows: intracranial hemorrhage (*p* = 0.808), mortality endpoint (*p* = 0.134), and revascularization (*p* = 0.824 Supporting information Table S5).

## 4 DISCUSSION

This meta‐analysis reports detailed analyses of 3 RCTs and 10 observational studies that compared GA with CS in patients with endovascular treatment for anterior circulation ischemic stroke. Our results indicated that CS was associated with improved functional outcome within 90 days, lower rates of intracranial hemorrhage, and mortality in the overall analysis and observational studies, compared with GA. However, for the RCTs, the rates of successful reperfusion and functional independence were higher in the GA group, with no differences between CS and GA in other secondary efficacy or safety outcomes.

Contrary to the four previous meta‐analysis (Brinjikji et al., 2017; Campbell et al., 2018; Gravel et al., 2018; Ilyas et al., 2018) that only have a single aspect of results from both RCTs and observational studies or RCTs, our meta‐analysis separately analyzed them and found that the inconsistencies were remarkable between the RCTs and the observational studies, further advancing the understanding of controversy in the choice of anesthesia methods during endovascular therapy for anterior circulation ischemic stroke. Both RCTs and observational studies have strengths and limitations that finally affect their results. Although RCTs could reduce the influence of confounders, they are usually small to modest sized (SIESTA (Schönenberger et al., 2016) 150 patients, ANSTROKE (Löwhagen Hendén et al., 2017) 90 patients, GOLIATH (Simonsen et al., 2018) 128 patients) and easily produce a highly selected patients to whom the endovascular therapy could be beneficial in terms of the specified selection criteria (Britton, McKee, Black, McPherson, & Sanderson, 1998) (only including patients for whom groin puncture could be performed within 6 hr from symptom onset and excluded patients with severe agitation (Schönenberger et al., 2016; Simonsen et al., 2018)). Moreover, the high rates of conversions from CS to GA in the RCTs (SIESTA (Schönenberger et al., 2016) 14.3% and GOLIATH (Simonsen et al., 2018) 15.6%), which might contribute to the worse outcome of CS patients demonstrated that the limitations of results also existed in RCTs. While observational studies may be more generalizable for all included patients (including patients within 8 hr from symptom onset to EVT and no limit to the NIHSS scores (Slezak et al., 2017)), thus, they are more powered to estimate the safety endpoints. Therefore, the results of the observational studies and RCTs are both valid to verify the effect of anesthesia methods on endovascular therapy.

The observed discrepancy in findings between the RCTs and the observational studies highlights the problem of confounders in terms of the different designs. Many points may be used to explain the inconsistencies: basic characteristics of patients, time to start EVT, and effect of anesthetic factors, thrombectomy devices, and the sites of occlusion.

Worse baseline conditions and vascular risk factors such as hypertension, heart disease, and hyperlipidemia were more obvious in GA within observational studies. For example, a large observational study, Slezak et al., 2017, found that the NIHSS score was higher in the CS group (*p* < 0.001), which contributed to the worse outcomes, but when adjusting the NIHSS score for the functional outcomes, the significance was lost. Although our subgroup analysis showed that there was no change in the results when adjusting the NIHSS scores, heterogeneity also existed in each method. However, for the three RCTs, the basic characteristics were well balanced between CS and GA except for one RCT, ANSTROKE (Löwhagen Hendén et al., 2017) which involved patients with higher NIHSS scores in GA leading to no difference in the outcomes between two groups. Thus, patients in good conditions with CS could obtain the better outcomes.

The sooner from onset of symptom to EVT, the better were functional outcomes. Even less than thirty minutes delayed may be evidently associated with poor functional outcome. In a meta‐analysis, Saver et al reported that among 1,000 patients receiving endovascular treatment, for every 15 min faster emergency department to endovascular therapy, an estimated 39 patients might have better outcomes after three months (Saver et al., 2016). In the observational studies, a longer treatment delay was more common in patients with GA (CS vs. GA: MD, −18.62, 95%CI, −33.98‐(−3.26), *p* = 0.018). While the time interval from onset to EVT between the GA and CS groups was remarkably consistent across the three RCTs (CS vs. GA: MD, −2.27, 95%CI, −17.58 to 13.04, *p* = 0.772). Therefore, when the time interval from symptom to endovascular treatment was similar, the superiority of GA was obvious.

The neuroprotective properties of anesthetic agents, hemodynamic effects, and vomiting during the anesthesia and activity and partnership of the department of anesthesiology need to be taken into account, despite there are not conclusive data. Different anesthesia factors between the RCTs and observational studies may contribute to the diverse outcomes. The Solitaire device, the modern technics, was associated with a higher rate of successful revascularization and a lower rate of symptomatic hemorrhage, in contrast to the first stent retriever (Campbell et al., 2016; Saber, Rajah, Kherallah, Jadhav, & Narayanan, 2018). However, except for the EKOS devices used in IMS II (Nichols et al., 2010), the other first thrombectomy devices were still used in many studies (Kim, Son, Kang, Hwang, & Kim, 2017; Lapergue et al., 2016). Thus, unlike the previous meta‐analysis, Ilyas et al. (2018); Gravel et al. (2018), only including the modern devices, some observational studies (Abou Chebl et al., 2015; Jumaa et al., 2010) in our study utilized the first devices. Although we performed subanalysis to adjust for the thrombectomy devices, the heterogeneity was difficult to be eliminated. Patients with the different sites of occlusion may be related to the various severity of stroke. Studies reported that the improved functional outcomes were more observed in the proximal arterial occlusion (Badhiwala et al., 2015). The functional outcomes were not adjusted for the sites of occlusion due to the lacking data.

Systematic bias should be critically considered in a meta‐analysis of published literature, and detection and adjustment for publication bias in statistical methods are common (Jin, Zhou, & He, 2015; Sedgwick, 2015). The fact that our meta‐analysis only included patients with anterior circulation ischemic stroke, which was different from the previous study, Brinjikji et al. (2017), with a mixture of patients with anterior and posterior circulation ischemic stroke, could reduce the bias of results. Egger tests revealed no relationship between the assessments of OR and study size for the most clinical outcomes.

Our conclusions are limited by variability in the designs and study reporting, which is inherent to meta‐analyses. Our meta‐analysis only included patients with anterior circulation stroke, which may lead to insufficiency of some data. Thus, some factors could not be evaluated, such as the usage of intra‐arterial thrombolytic agents and hemodynamics during anesthesia.

## 5 SUMMARY

Our meta‐analysis indicated that the results of the observational studies were in contrast to that of RCTs. By analyzing the inconsistencies, we found that patients with CS were associated with the improved functional outcomes when patients were in good conditions, but when the basic features and the time interval from onset to EVT were well balanced, the results were opposite. Thus, large‐scale RCTs are required to fully elucidate what factors could influence the effects of the two anesthesia methods on the clinical outcomes during endovascular treatment for anterior circulation ischemic stroke.

## COMPETING INTERESTS

All authors declare no competing interests.

## DISCLOSURES

All authors report no disclosures.
