# Supplementary material for: Anesthesia for endovascular treatment in anterior circulation stroke: A systematic review and meta‐analysis
Source: Brain Behav. 2018 Dec 3;9(1):e01178. doi: 10.1002/brb3.1178 (PMC6346417; doi:10.1002/brb3.1178)

**eFigure Legends**

**eFigure 1**. Risk of bias summary for randomized controlled trials (RCTs), using the Cochrane Collaboration’s tool. “+” represents low risk and “？” represents unclear.

**eFigure 2.** System bias for all clinical outcomes in Figure 2.

**eTable 1.** Pooled demographic characteristics.

| Pooled categorical data, No. (%) | All Studies | |  |  |  | Observatioanl Studies | | |  |  | | RCTs | | |  | |  | |
| --- | --- | --- | --- | --- | --- | --- | --- | --- | --- | --- | --- | --- | --- | --- | --- | --- | --- | --- |
| Characteristic | CS | GA | p | Studies | | CS | GA | p | Studies | | CS | | GA | p | | Studies | |  |
| Patients NO. | 1624 | 1473 |  |  | | 1439 | 1290 |  |  | | 185 | | 183 |  | |  | |  |
| Men | 508  /990  (51.3) | 459  /875  (52.5) | 0.622 | 9 | | 413  /805  (51.3) | 349  /692  (50.4) | 0.737 | 6 | | 95  /185  (51.4) | | 110  /183  (60.1) | 0.091 | | 3 | |  |
| Hypertension | 440  /780  (56.4) | 460  /743  (61.9) | 0.029 | 7 | | 332  /595  (55.8) | 341  /560  (60.9) | 0.079 | 4 | | 108  /185  (60.0) | | 119  /183  (65.0) | 0.190 | | 3 | |  |
| Atrial fibrillation | 305  /917  (33.3) | 273  /822  (33.2) | 0.983 | 8 | | 224  /732  (30.6) | 195  /639  (30.5) | 0.973 | 5 | | 81  /185  (43.8) | | 78  /183  (42.6) | 0.822 | | 3 | |  |
| Heart disease | 90  /535  (16.8) | 107  /454  (23.6) | 0.008 | 4 | | 64  /413  (15.5) | 81  /336  (24.1) | 0.003 | 2 | | 26  /122  (21.3) | | 26  /118  (22.0) | 0.892 | | 2 | |  |
| Hyperlipidemia | 206  /634  (32.5) | 199  /545  (36.5) | 0.147 | 5 | | 175  /512  (34.2) | 174  /427  (40.7) | 0.038 | 3 | | 31  /122  (25.4) | | 25  /118  (21.2) | 0.439 | | 2 | |  |
| Diabetes mellitus | 192  /917  (20.9) | 188  /820  (22.9) | 0.317 | 8 | | 159  /732  (21.7) | 153  /637  (24.0) | 0.312 | 6 | | 33  /185  (17.8) | | 35  /183  (19.1) | 0.750 | | 3 | |  |
| Smoking | 90  /320  (28.1) | 144  /449  (32.1) | 0.241 | 4 | | 49  /135  (36.3) | 111  /266  (41.7) | 0.294 | 1 | | 41  /185  (22.2) | | 33  /183  (18.0) | 0.323 | | 3 | |  |
| Site of occlusion |  |  |  | 9 | |  |  |  | 6 | |  | |  |  | | 3 | |  |
| ICA | 182  /990  (18.4) | 271  /875  (31.0) | 0.000 |  | | 150  /805  (18.0) | 241  /692  (34.8) | 0.000 |  | | 32  /185  (17.3) | | 30  /183  (16.4) | 0.817 | |  | |  |
| MCA | 756  /990  (76.4) | 548  /875  (62.6) | 0.000 |  | | 644  /805  (71.4) | 443  /692  (64.0) | 0.000 |  | | 112  /185  (60.5) | | 105  /183  (57.4) | 0.537 | |  | |  |
| Others | 51  /990  (5.2) | 52  /875  (5.9) | 0.455 |  | | 11  /805  (1.4) | 8  /692  (1.2) | 0.717 |  | | 40  /185  (21.6) | | 44  /183  (24.0) | 0.580 | |  | |  |
| Pooled continuous data | MD | 95%CI | p |  | | MD | 95%CI | p |  | | MD | | 95%CI | p | |  | |  |
| Age | 2.03 | 0.18-  3.88 | 0.031 | 8 | | 3.29 | 1.21-5.38 | 0.002 | 5 | | -0.37 | | (-2.54)-1.81 | 0.740 | | 3 | |  |
| ASPECTs | 0.003 | -0.09-  0.10 | 0.944 | 4 | | 0.02 | -0.22  -0.26 | 0.857 | 2 | | 0.000 | | -0.10-  0.10 | 1.000 | | 2 | |  |
| Baseline NIHSS | -1.86 | -3.27  -(-1.68) | 0.000 | 10 | | -1.96 | -2.90-(-1.70) | 0.000 | 7 | | -1.57 | | -3.07-  (-0.06) | 0.041 | | 3 | |  |
| Time from stroke onset | | | |  | |  |  |  |  | |  | |  |  | |  | |  |
| To start EVT | -11.18 | -22.10-  (-0.26) | 0.045 | 7 | | -18.62 | -33.98-(-3.26) | 0.018 | 4 | | -2.27 | | -17.58-  (13.04) | 0.772 | | 3 | |  |
| To reperfusion | -0.21 | -11.32-10.90 | 0.971 | 5 | | -16.20 | -38.55-6.15 | 0.155 | 2 | | 4.82 | | -7.49-17.12 | 0.443 | | 3 | |  |
| Procedure time | 1.44 | -6.01-8.89 | 0.705 | 5 | | 2.13 | -5.62-9.88 | 0.590 | 3 | | 4.59 | | -17.88  -27.07 | 0.689 | | 2 | |  |

Abbreviations: RCTs, randomized controlled trials; ICA, internal carotid artery; MCA, middle cerebral artery; ASPECTs, Alberta Stroke Program Early Computed Tomography Score; NIHSS, The National Institutes of Health Stroke Scale; EVT, endovascular treatment; MD, mean difference; CI, confidence interval.

**eTable 2**. Quality of observational studies.

| Author | Selection | Comparability | Outcome |
| --- | --- | --- | --- |
| Van den Berg et al, 2015 | **** | * | *** |
| Slezak et al, 2017 | **** |  | *** |
| Matthew K Whalin et al, 2013 | **** |  | *** |
| Jumaa et al, 2010 | ** |  | ** |
| Alex Abou-Chebl et al, 2014 | * |  | *** |
| Alex Abou-Chebl et al, 2010 | **** | * | *** |
| John et al, 2017 | ** |  | ** |
| Barcard et al, 2017 | **** | * | *** |
| HERMES,ISC, 2017 | **** |  | *** |
| Shan et al, 2018 | **** | * | *** |

Newcastle-Ottawa Scale for the assessment of observational studies. One star represents each quality item, with 4 stars as the maximum for selection of groups, 2 stars as the maximum for comparability, and 3 stars as the maximum for outcome.

**eTable 3.** Meta-regression analyses for functional independence.

|  | All Studies | | | |  | | Observational Studies | | | |
| --- | --- | --- | --- | --- | --- | --- | --- | --- | --- | --- |
| Characteristics | Adjusted OR | 95%CI | p | p for heterogeneity | | Adjusted OR | | 95%CI | p | p for heterogeneity |
| NO. of patients | 1.34 | 1.06-1.69 | 0.016 | 0.000 | | 1.67 | | 1.49-1.87 | 0.000 | 0.830 |
| Time to start EVT | 1.19 | 0.82-1.73 | 0.360 | 0.000 | | 1.72 | | 1.37-2.16 | 0.000 | 0.895 |

Abbreviations: OR, odds ratio; CI, confidence interval; EVT, endovascular treatment

**eTable 4.** Clinical Outcomes in RCTs and observational studies.

|  | Observational studies | | | |  |  | RCTs | | | | |
| --- | --- | --- | --- | --- | --- | --- | --- | --- | --- | --- | --- |
|  | CS | GA | Odds ratio  (95% CI) | P value | I2 | | CS | GA | Odds ratio (95% CI) | P value | I2 |
| Functional Independence | 836/1944  (43.0%) | 470/1545  (30.4%) | 1.79  (1.42,2.24) | 0.000 | 15% | | 65/185  (35.1%) | 90/183  (49.2%) | 0.55  (0.34,0.89) | 0.010 | 15% |
| Revascularization | 357/699  (51.1%) | 374/594  (63.0%) | 0.94  (0.65,1.36) | 0.580 | 67% | | 140/185  (75.7%) | 156/183  (85.2%) | 0.51  (0.30,0.89) | 0.020 | 0% |
| Intracranial Hemorrhage | 365/1261  (28.9%) | 378/1102  (34.3%) | 0.71  (0.51,0.98) | 0.002 | 47% | | 6/108  (5.6%) | 6/110  (5.5%) | 1.40  (0.10,19.91) | 0.800 | 63% |
| Pneumonia | 57/498  (11.4%) | 35/282  (12.8%) | 0.69  (0.34,1.41) | 0.310 | 42% | | 10/122  (8.2%) | 16/118  (13.6%) | 0.57  (0.13,2.6) | 0.470 | 66% |
| Intraprocedural Complications | 47/486  (9.7%) | 30/409  (7.3%) | 1.04  (0.58,1.89) | 0.940 | 10% | | 2/77  (2.6%) | 1/73  (1.4%) | 1.92  (0.17,21.64) | 0.600 | - |
| Mortality at 90 d | - | - | 0.63  (0.50, 0.78) | 0.000 | 0% | | - | - | 1.30  (0.76,2.22) | 0.330 | 0% |

Abbreviations: CS, conscious sedation; GA, general anesthesia; RCTs, randomized controlled trials; CI, confidence interval.

**eTable 5.** Bias for clinical outcomes using Egger test

| Outcomes | All studies  (p value) | Observational studies  (p value) | RCTs  (p value) |
| --- | --- | --- | --- |
| Functional independence | 0.564 | 0.976 | 0.760 |
| Revascularization | 0.824 | 0.842 | 0.341 |
| Intracranial Hemorrhage | 0.808 | 0.455 | - |
| Pneumonia | 0.572 | 0.724 | - |
| Intraprocedural Complications | 0.769 | 0.906 | - |
| Mortality | 0.134 | 0.573 | 0.277 |

**eFigure 1.** Risk bias for RCTs


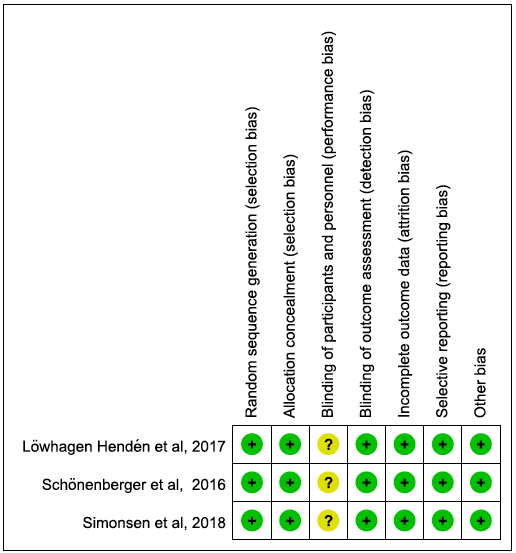


**eFigure 2.** Funnel plot for clinical outcomes in Figure 2


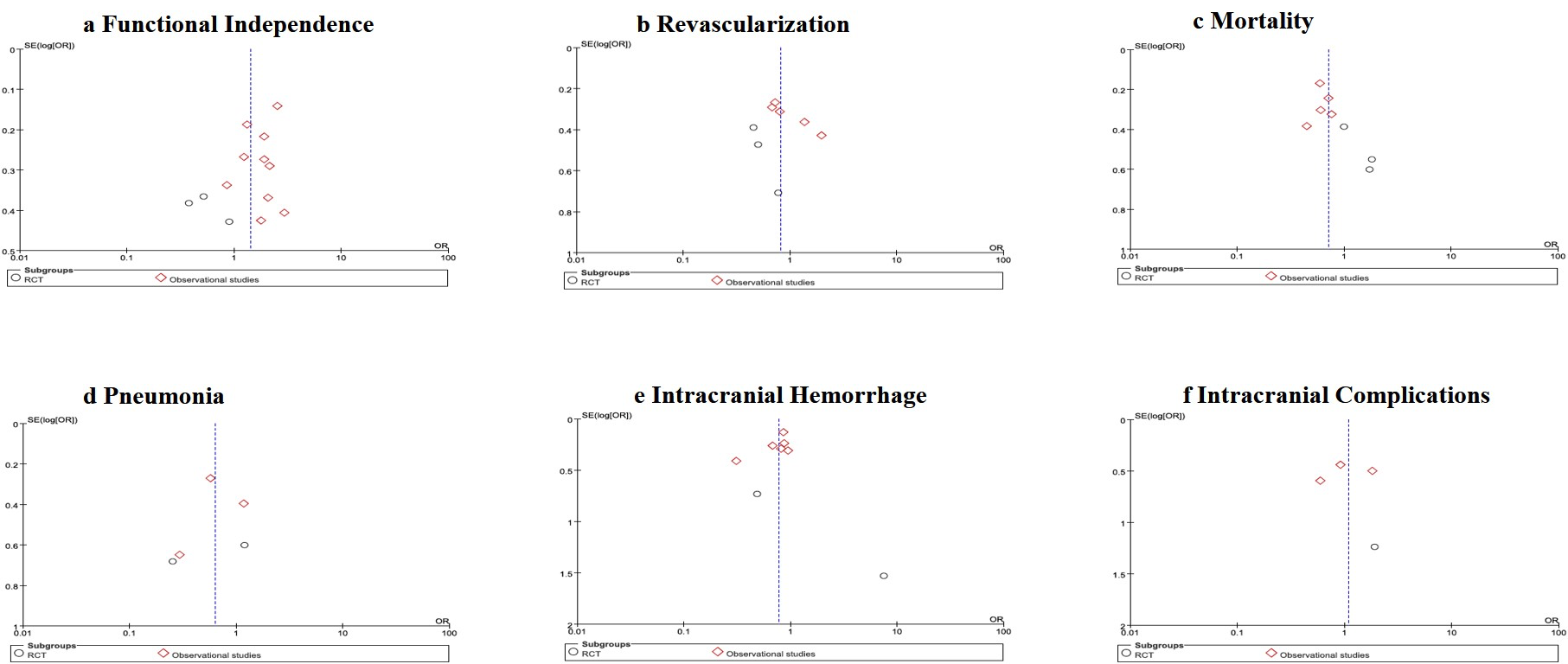

Supplement: Supplementary file 1 [file BRB3-9-e01178-s001.docx]
